# T Helper Cell Subsets Specific for *Pseudomonas aeruginosa* in Healthy Individuals and Patients with Cystic Fibrosis

**DOI:** 10.1371/journal.pone.0090263

**Published:** 2014-02-27

**Authors:** Hannah K. Bayes, Stephen Bicknell, Gordon MacGregor, Tom J. Evans

**Affiliations:** 1 Institute of Infection, Immunology and Inflammation, College of Medical, Veterinary and Life Sciences, University of Glasgow, Glasgow, United Kingdom; 2 West of Scotland Cystic Fibrosis Centre, Gartnavel General Hospital, Glasgow, United Kingdom; University of Tübingen, Germany

## Abstract

**Background:**

We set out to determine the magnitude of antigen-specific memory T helper cell responses to *Pseudomonas aeruginosa* in healthy humans and patients with cystic fibrosis.

**Methods:**

Peripheral blood human memory CD4^+^ T cells were co-cultured with dendritic cells that had been infected with different strains of *Pseudomonas aeruginosa*. The T helper response was determined by measuring proliferation, immunoassay of cytokine output, and immunostaining of intracellular cytokines.

**Results:**

Healthy individuals and patients with cystic fibrosis had robust antigen-specific memory CD4^+^ T cell responses to *Pseudomonas aeruginosa* that not only contained a Th1 and Th17 component but also Th22 cells. In contrast to previous descriptions of human Th22 cells, these Pseudomonal-specific Th22 cells lacked the skin homing markers CCR4 or CCR10, although were CCR6^+^. Healthy individuals and patients with cystic fibrosis had similar levels of Th22 cells, but the patient group had significantly fewer Th17 cells in peripheral blood.

**Conclusions:**

Th22 cells specific to *Pseudomonas aeruginosa* are induced in both healthy individuals and patients with cystic fibrosis. Along with Th17 cells, they may play an important role in the pulmonary response to this microbe in patients with cystic fibrosis and other conditions.

## Introduction

Cystic fibrosis (CF) typically results in recurrent pulmonary infections and inflammation that produces progressive respiratory failure [1], [2]. The most common pathogen in these patients is *Pseudomonas aeruginosa* (PA), which produces a neutrophil dominated host response that does not, however, clear the infection [3]. Indeed, this persistent inflammatory response contributes to progressive lung injury [4], [5] and thus pulmonary immune responses to *P. aeruginosa* are a potential therapeutic target.

Colonization with this bacterium results in an antibody response, which is not, however, protective [6]. In contrast, cell-mediated immunity to *P. aeruginosa* is important in host defence [7]. Recently, attention has focused on the novel T helper 17 (Th17) subset of Th cells [8]. These produce the signature cytokine IL-17 that plays a critical role in the generation and recruitment of neutrophils to sites of infection. In experimental murine models of acute pulmonary *P. aeruginosa* infection, there is evidence that Th17 cells play a key role in vaccine-induced protection [9]. Human studies have shown IL-17 is produced in CF lung, and that Th17 cells are present in the submucosa of airways from CF patients, although IL-17 is also produced from innate immune cells [10], [11]. Humans typically have a very strong Th17 memory response to *Candida albicans* as well as *Staphylococcus aureus* [12], [13]. However, it is not known whether there are similar memory responses to PA or whether these are altered in patients with CF.

IL-17 is commonly co-expressed with IL-22 in classic Th17 cells. IL-22 is a cytokine of the IL-10 family that has important effects on non-immune cells, promoting repair of epithelial surfaces and inducing an anti-microbial state [14]. More recently, a distinct lineage of Th cells producing IL-22 in the absence of IL-17 has been discovered, termed Th22 cells [15]–[18]. These cells have been found to possess a skin homing phenotype and infiltrate the skin in inflammatory disorders such as psoriasis in which IL-22 has been proposed to play a pathogenic role. In contrast, IL-22 also induces a variety of antimicrobial peptides and is important in host defence against pulmonary pathogens such as *Klebsiella pneumonia* [19]. Additionally, in a murine model of gut inflammation following infection with *Citrobacter rodentium*, IL-22 plays an important role in host defence [20], [21]. In this model, IL-22 is produced both by innate lymphoid cells as well as Th22 cells [18]. IL-22 is also required to prevent dissemination of the commensal enteric bacteria from the genus *Alcaligenes* [22]. As yet, no specific pathogen or antigen has been demonstrated to elicit a human memory Th22 response.

Given that IL-22 promotes epithelial repair and induces an antimicrobial state, we hypothesised that Th22 cells could play an important role in host defence against lung infection with *P. aeruginosa* in CF and other clinical settings. Additionally, given the proposed role of Th17 cells in protection against PA infection, we hypothesized that PA-specific Th17 cells would be found in patients with CF. In order to test these hypotheses, we set out to examine the Th22, Th17 and Th1 cell responses to *P. aeruginosa* in both control and healthy individuals as well as patients with cystic fibrosis. We found that both healthy individuals as well as those with cystic fibrosis had robust antigen-specific Th17, Th1 and Th22 responses to *P. aeruginosa*. The PA-specific Th22 cells were CCR6^+^ but lacked the skin-homing receptors CCR4 and CCR10 that have previously been found on human Th22 cells. PA-specific Th17 cells were reduced in patients with CF. These PA-specific Th cell subsets represent novel effector cells that can modulate the host anti-bacterial and inflammatory response to this pathogen and hence could be targets for novel therapeutic interventions in PA infection in CF and other conditions.

## Materials and Methods

### Ethics

All subjects gave informed written consent. The study was approved by West of Scotland Research Ethics Committee Number 2, a committee of the West of Scotland Research Ethics Service (WoSRES) that oversees ethical review of studies carried out on patients of the National Health Service Greater Glasgow and Clyde Health Board.

### Participants and Cell Isolation

Peripheral blood samples were collected from adult CF patients attending the West of Scotland Adult CF Unit, Glasgow. Healthy individuals (controls) had no history of respiratory disease or inter-current illness. Participant characteristics are shown in Table 1. Patients with CF attending the West of Scotland Adult CF Unit are deemed to be chronically colonized with *Pseudomonas aeruginosa* if sputum cultures remain positive for the organism after two attempts to clear the organism with combination antibiotic eradication therapy. Intermittent infection is deemed to exist when PA has been isolated and eradicated via antibiotic therapy.

**Table 1:** Characteristics of participants providing peripheral blood specimens for the analysis of T helper cell responses to *Pseudomonas aeruginosa*.

	Patients with cystic fibrosis	Healthy controls
Participants (n)	8	10
Age, median (IQR), years	23 (23.5–30)	26 (24–29)
Male sex, n (%)	5 (62.5)	4 (40)
Chronic *Pseudomonas aeruginosa* colonization, n	7	na
FEV_1_ median (IQR), % predicted	38.2 (35.2–44.4)	na
No. of patients with respiratory exacerbation at time of sample collection, n (%)	6 (75)	na

n, number in group; na, non-applicable; IQR, inter-quartile range; FEV_1_, forced expiratory volume in 1 second.

PBMCs were obtained by Ficoll-Paque gradient centrifugation (GE Healthcare). CD14^+^ monocytes were then magnetically isolated by a positive selection kit (Miltenyi Biotech). Memory CD4^+^ T cells (purity for CD4^+^CD45RO^+^ >98%) were magnetically isolated by a negative selection kit (Miltenyi Biotech). Memory CD4^+^ T cells were further sorted into CCR6-enriched and CCR6-depleted populations using positive selection microbeads (Stemcell Technologies). Proliferation was measured with CFSE (carboxyfluorescein diacetate succinimidyl ester; Invitrogen) or proliferation dye eFluor®450 (eBioscience) incorporated prior to cell culture.

### Bacteriology

Laboratory *P. aeruginosa* (PA) strains PA103ΔpcrV and PA103ΔUΔT [23], [24] were kind gifts of Dr. Dara Frank, University of Wisconsin. Both strains have modulation of the function of the type three secretion system (T3SS), a common finding amongst CF PA strains; PA103ΔpcrV lacks the pore-forming protein of the T3SS rendering it non-functional and PA103ΔUΔT lacks the major translocation proteins (exoU and exoT) of the T3SS. Clinical non-mucoid PA strains Yorkhill 1 and Yorkhill 2, and clinical mucoid PA strain Yorkhill 5 were from CF samples (provided by Dr Craig Williams, Royal Hospital for Sick Children, Yorkhill). PA strains were grown to mid-log growth phase prior to infection.

### Cell Culture

Dendritic cells (DCs) were derived from CD14^+^ monocytes with IL-4 (500 IU/mL; Peprotech) and GM-CSF (50 ng/mL; Peprotech) for 7-days. DCs were infected with live PA strains as indicated. Ninety-minutes following infection, DCs were treated with bactericidal antibiotics (100 U/mL penicillin-streptomycin and 10 mg/mL gentamicin; both Sigma) followed by overnight incubation. Additionally, in some experiments, DCs were treated with tetanus toxoid (5 ug/mL; Calbiochem) or heat-killed preparation of Candida albicans (InvivoGen).

Infected DCs (1×10^4^ cells) were cultured with autologous memory CD4^+^ T cells (1×10^5^ cells) in 96-well U-bottom plates in IMDM+Glutamax medium (Gibco Life Technologies) supplemented with 5% fetal bovine serum (Invitrogen) and 100 U/mL penicillin-streptomycin for 6-days. For polyclonal stimulation conditions, CD4^+^ T cells were cultured with plate-bound anti-CD3 (5 ug/mL) and soluble anti-CD28 (1 ug/mL) (both eBioscience).

### Measurement of CD4^+^ T cell response

Following 6-days of co-culture, supernatants were harvested and cytokine levels measured via ELISA for IL-17A, IL-22, and IFN-γ (eBioscience).

### Cell Staining and Analysis

Cells were stimulated for 5-hours with phorbol 12-myristate 13-acetate (50 ng/ml; Sigma) and ionomycin (500 ng/ml; Sigma) in the presence of Golgiplug (1 µl/ml; BD Pharmingen) at 37°C. Cells were fixed using 4% paraformaldehyde (VWR International) and made permeable, following extracellular staining, using Perm/Wash (BD Biosciences).

Extracellular staining was with phycoerythrin-labelled anti-CD4 (RPA-T4; eBioscience), phycoerythrin-labelled anti-αβTCR (IP26; eBioscience), phycoerythrin-Cy7-labelled anti-CCR4 (IGI; BD Pharminogen), AlexFluor®647-labelled anti-CCR9 (BL/CCR9; BioLegend), phycoerythrin-labelled anti-CCR10 (FAB 3478P; R&D), CD86 and CD40. Intracellular staining was with eFluor®660-labelled anti-IL-22 (22URTI; eBioscience), phycoerythrin-labelled anti-IL-22 (BG/IL-22; Biolegend), PerCP-Cy5.5-labelled anti-IFN-γ (4S.B3; eBioscience), and AlexFluor®488 anti-IL-17A (eBioDEC17; eBioscience). Corresponding isotype controls were used. Dead cell exclusion was achieved either by Boolean-gating based on cell scatter properties or labelling with Fixable Viability Dye eFluor®506 (eBioscience). Stained cells were analysed on either a FACSCalibur or CyAn flow cytometer (BD Biosciences). Collected data were analysed using FlowJo software (Tree Star).

### Statistical analysis

Non-parametric statistical tests were used (Mann-Whitney, Kruskal-Wallis (KW), and, where appropriate, Dunn's multiple comparison test), using Prism Version 6.0 (GraphPad Software).

## Results

### Antigen-specific memory CD4^+^ T cell responses exist against *P. aeruginosa* in Healthy Individuals

**Figure 1:**
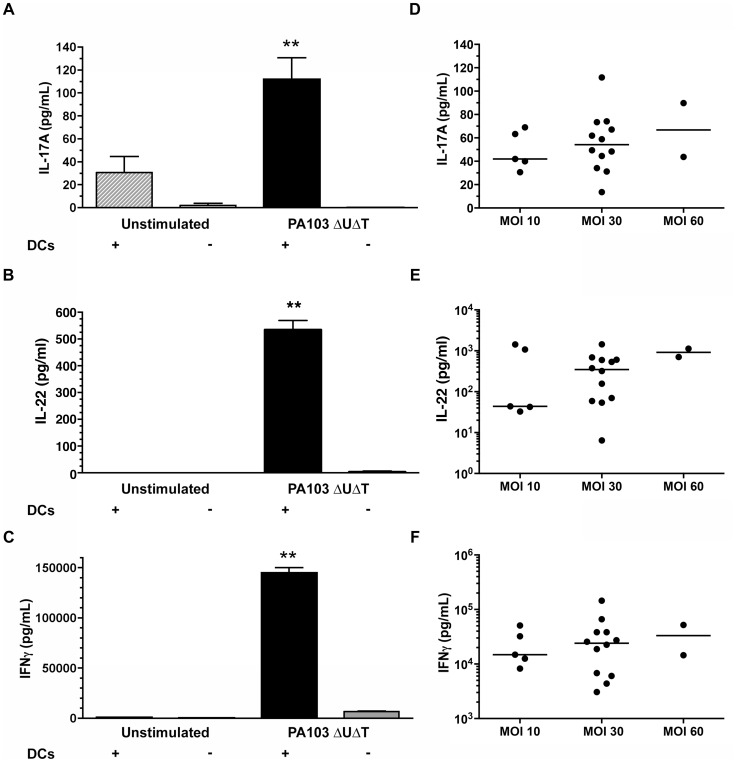
Cytokine production of human memory CD4^+^ T cells in response to *P. aeruginosa*. **A–C** Sorted human memory CD4^+^ T cells from healthy volunteers were co-cultured either with dendritic cells that had been infected with *P. aeruginosa* (DCs +) or with the supernatant from such infected DCs (DC −). Levels of IL-17A (**A**), IL-22 (**B**) and IFN-γ (**C**) were measured in supernatants after 6 days. DCs were infected at a MOI of 60 before culturing with T cells. Columns show the mean of triplicate determinations; error bars are ±1 standard error of mean. Results are representative of experiments in three separate individuals. **D–F**, secreted cytokine levels following DC infection at a variety of different MOIs. Each point represents a value from an individual healthy individual.

In order to examine antigen-dependent T cell memory responses from healthy controls and patients with CF, we isolated monocyte-derived dendritic cells (DCs) and CD4^+^CD45RA^−^CD45RO^+^ memory T cells from peripheral blood. Memory CD4^+^ T cells were co-cultured with either uninfected DCs, DCs infected with PA, or the supernatant of such DCs, and the production of IL-17A, IL-22 and IFN-γ measured in the culture supernatant by ELISA (Figure 1).

Firstly, we examined the memory T cell responses of healthy controls. Small amounts of IL-17A were produced by T cells cultured with unstimulated DCs; this was abolished in the absence of DCs (Figure 1A). In the absence of DC infection, there was no significant production of either IL-22 (Figure 1B) or IFN-γ (Figure 1C). In contrast, co-culture of memory T cells with DCs infected with PA103 gave robust production of IL-17A, IL-22 and IFN-γ (Figure 1). This was almost completely abolished when T cells were cultured with infected DC supernatants alone (Figure 1); thus direct contact between T cells and DCs is required for cytokine production. No cytokines were produced when T cells were infected directly in the absence of DCs or when infected DCs were cultured alone in the absence of T cells (data not shown). Thus, the observed memory Th cell responses in healthy individuals were entirely dependent on physical contact between the DCs and T cells, as expected of an antigen-driven response. Cytokine production did not vary significantly following co-culture of memory T cells with DCs infected at multiplicities of infection (MOIs) between 10–60 (Figure 1D–F), suggesting even at the lowest MOI used the stimulating effect on memory T cells was already maximal.

Next, we compared this cytokine production by memory CD4^+^ T cells in healthy individuals against different strains of PA. Similar levels of these three cytokines were produced following infection of DCs with strains of PA103 that differed in the possession of a functional type III apparatus (Figure S1). Additionally, these cytokine levels did not vary significantly between these laboratory strains and the response to PA strains isolated from patients with CF (Figure S1).

**Figure 2:**
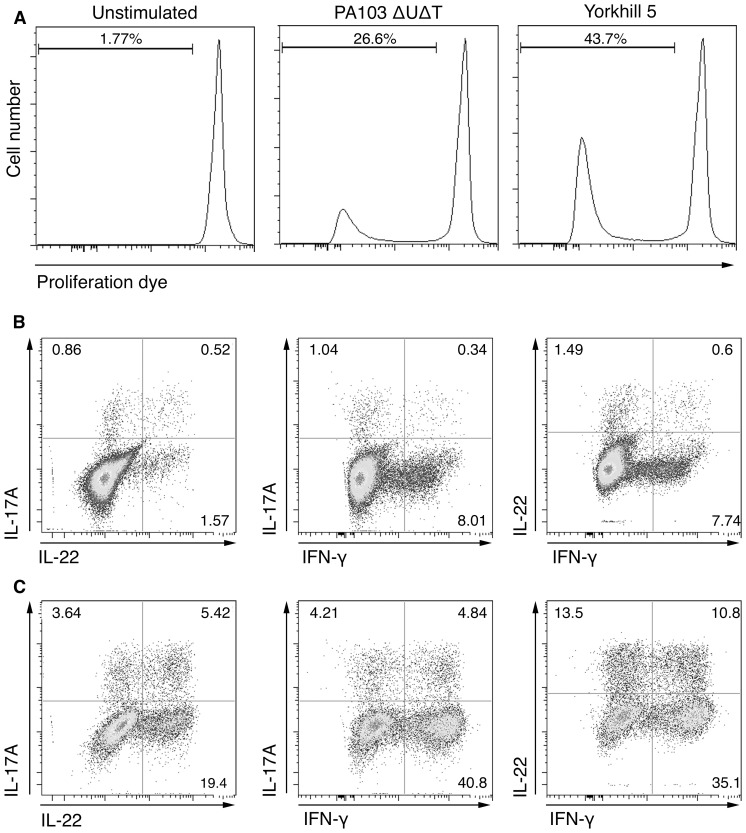
Cell proliferation of memory CD4^+^ T cells following co-culture with infected dendritic cells (DCs). (**A**) Memory CD4^+^ T cells loaded with proliferation dye were co-cultured with unstimulated DCs, DCs infected with PA103ΔUΔT MOI 30 (PA103), or DCs infected with Yorkhill 5 MOI 30 (Yorkhill 5). Panels show the distribution of the proliferation dye fluorescence in the cell population after 6-days; inset figures show the percentage of the initial population that has undergone one or more cell divisions. (**B**) Patterns of cytokine expression by non-proliferating CD4^+^ T cells following 6-days of culture with DCs infected with Yorkhill 5. (**C**) Patterns of cytokine expression by CD4^+^ T cells proliferating in response to culture with DCs infected with Yorkhill 5. Numbers in plot represent per cent cells in each quadrant. The experiment was repeated in 3 independent individuals with the same result.

We then examined the proliferation of memory CD4^+^ T cells of healthy individuals following co-culture with DCs alone or DCs infected with PA, using a flow cytometric method. As expected, little proliferation was seen with T cells cultured with uninfected DCs (Figure 2A). However, marked proliferation of CD4^+^ T cell was seen in response to DCs infected with either laboratory PA103ΔUΔT (average proliferation [+/−SEM] 18.8% [+/−9.0%] for three donors) and clinical Yorkhill 5 (average proliferation [+/−SEM] 35.1% [+/−5.2%] for three donors) PA strains (Figure 2A). We also stained these cells for the presence of intracellular cytokines to determine which cells were producing the measured cytokines. IL-17 and IL-22 production in the co-cultures was predominantly by cells proliferating in response to PA (Figure 2B and C). Proliferating cells producing intracellular IL-17 and/or IL-22 were also confirmed to be αβ T cell receptor positive cells (data not shown). No intracellular cytokines were produced in DCs. Small amounts of intracellular IFN-γ were expressed by the non-proliferating CD4^+^ memory cells following polyclonal stimulation; however, IFN-γ was only secreted from memory CD4^+^ T cells in response to PA infected DCs (Figure 1). Collectively, these data show the existence of a classical antigen-specific memory CD4^+^ T cell response to *P. aeruginosa* in healthy individuals with no previous history of pulmonary infection with this organism.

### DC Activation following Infection with *P. aeruginosa*

**Figure 3:**
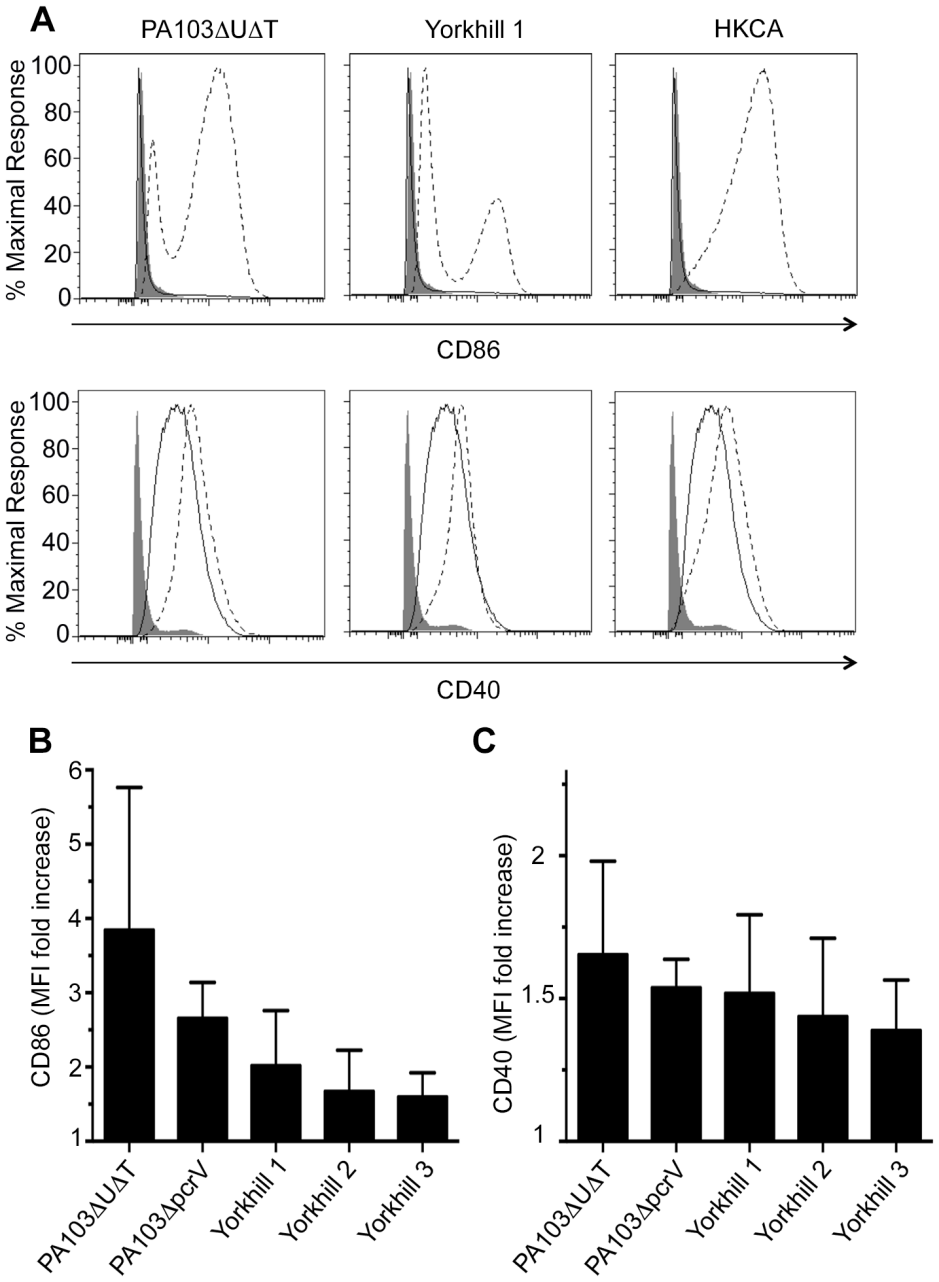
DC Activation following infection with different strains of *P. aeruginosa*. Levels of CD86 (**A**) and CD40 (**B**) following DC activation 24 hours following treatment with the indicated microbes. The panels show representative plots of isotype staining (solid shading), staining of unstimulated DCs (solid line) and staining following microbial treatment (dotted line). **B** and **C**, mean fold-increase in mean fluorescence intensity (MFI) of CD86 and CD40 respectively in 5 independent individuals. This was calculated as the fold-increase in MFI in infected DCs versus uninfected controls. Columns show mean and SEM.

To explore potential differential effects of the different PA strains on DC activation, we stained DCs for the activation markers CD86 and CD40 following infection with the different PA strains (Figure 3). Figure 3A shows representative plots of flow cytometric analysis of CD86 and CD 40 on the surface of DCs following infection with PA103ΔUΔT and Yorkhill 1 strains of PA as well as exposure to heat killed *Candida albicans*. These examples all show a marked upregulation of these activation markers on the surface of DCs. This was quantified for both CD86 and CD40 for a range of strains as shown in Figure 3B and 3C respectively. All strains tested produced an increase in expression of these markers. There was a trend towards slightly higher levels of expression in DCs infected with the PA103 strains, but this did not reach statistical significance.

### Cytokine secretion by *P. aeruginosa*-specific CD4^+^ T cells in patients with CF and controls

**Figure 4:**
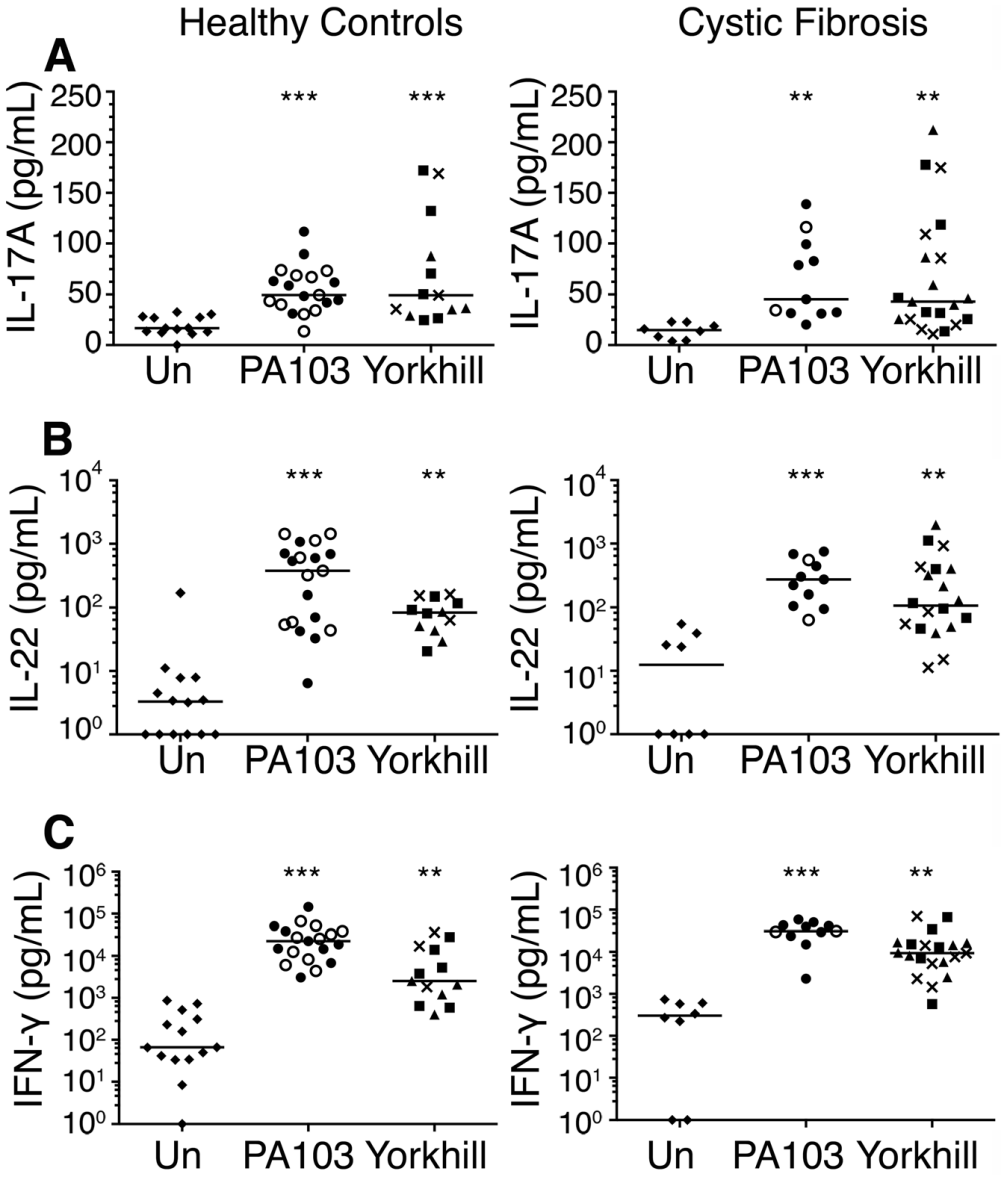
Cytokine production by human memory CD4^+^ T cells in healthy controls and cystic fibrosis. Human memory CD4^+^ T cells were exposed to unstimulated DCs or DCs infected with the laboratory strains (PA103) or clinical *Pseudomonas aeruginosa* strains (Yorkhill). After 6 days of co-culture levels of (**A**) IL-17, (**B**) IL-22 and (**C**) IFN-γ were measured. Each point represents the mean of triplicate wells and is the result from one individual. The different bacterial strains used are: ♦, unstimulated; ○, PA103 ΔpcrV; •, PA103ΔUΔT; ▴, YH1; ▪, YH2; ×, YH5. The line indicates the median value. Differences between infection conditions (**A**–**C**) were evaluated by a Kruskal-Wallis test all of which had p values <0.0009). Differences between unstimulated and infected conditions were then tested using Dunn's multiple comparison test. ** significant p<0.01 and *** significant p<0.001.

We then examined the cytokine responses of peripheral memory T cells in response to PA infected DCs in patients with CF compared to healthy individuals. All CF patients had a history of chronic or intermittent PA infection (Table 1). As seen in the experiments with T cells from healthy control individuals, memory CD4^+^ T cells from patients with CF produce significant amounts of IL-17A, IL-22 and IFN-γ in response to DC infection with either laboratory or clinical PA strains compared with uninfected DCs (Figure 4A, B and C). However, the magnitude of this response between patients with CF with a history of *P. aeruginosa* infection and healthy controls was not significantly different for all three of the cytokines measured (p>0.05 for all strains, Kruskal Wallis).

### *P. aeruginosa*-specific Th22, Th17 and Th1 responses in patients with CF and controls

**Figure 5:**
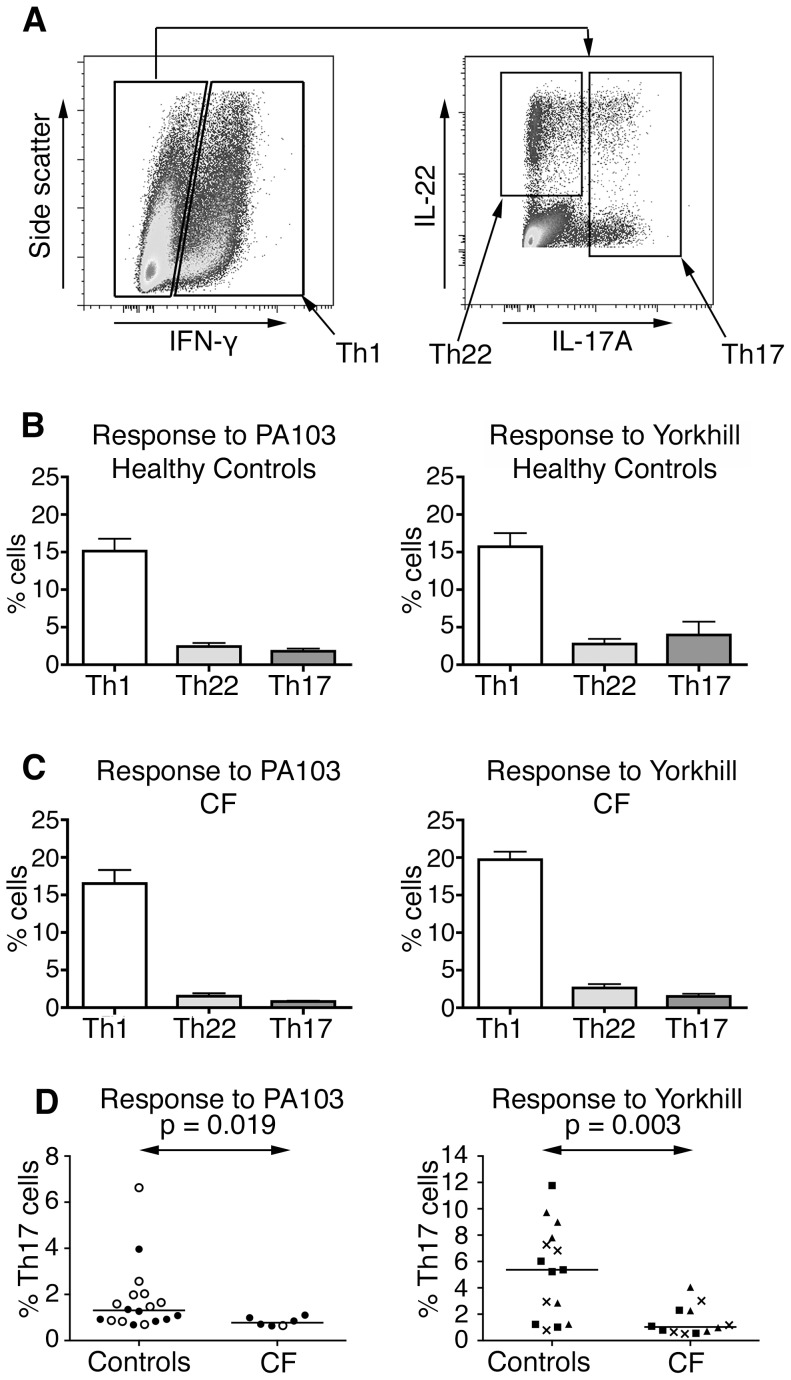
Memory CD4^+^ T cells subset response to different Pseudomonas strains in CF and controls. Following co-culture with dendritic cells infected with laboratory Pseudomonas aeruginosa (PA) strains (PA103) or clinical PA strains (Yorkhill), CD4^+^ T cells were classified into T helper cell subsets based on intracellular cytokine straining patterns, as shown for a representative patient in panel **A**. The Th1, Th17 and Th22 responses to both PA103 and Yorkhill PA strains is shown for healthy controls (**B**) and patients with CF (**C**), as a percentage of the total numbers of CD4^+^ memory cells analysed. The columns show the mean values +/− SEM; n = 8 for CF patients and 10 for healthy controls. Panel **D** shows the proportions of PA-specific Th17 cells in CF patients compared with controls. Each point represents the result from one individual; different bacterial strains are indicated by the symbols as described in Figure 4. The line indicates the median value. Differences between controls and patients with CF (**D**) were evaluated by a Mann-Whitney test.

Further to assess the Th cell subsets constituting the memory CD4^+^ T cell response to PA, we used polyclonal re-stimulation and intracellular staining of the resultant CD4^+^ T cell populations present at the end of the co-cultures followed by flow cytometric analysis. Th1 cells are characterized by their production of IFN-γ on antigen stimulation. In humans, Th1 cells can co-produce IL-17 and IL-22, which was indeed initially classed as a Th1 cytokine [25]. To provide an unambiguous classification of the different cytokine producing cells, we adopted the following definitions. CD4^+^ T cells expressing IFN-γ were designated as Th1 cells; Th17 cells were any IFN-γ negative cell producing IL-17A with/without IL-22; and Th22 cells were defined as those expressing IL-22 alone (Figure 5A). We used this gating strategy to quantify the proportions of memory CD4^+^ T cells in each of these categories following stimulation with PA-infected DCs.

In healthy controls (Figure 5B) and patients with CF (Figure 5C) the predominant responding population was a memory Th1 response. Within the population of IFN-γ positive cells proliferating in response to PA infected DCs, there were cells that that co-produced IL-17 and/or IL-22 (Figure 2C). Significantly, however, there were clear populations of Th22 and Th17 cells, as defined above, i.e. IFN-γ^−^ IL-17^−^ IL-22^+^ and IFN-γ^−^ IL-17^+^ respectively (Figure 5). The proportion of Th1 and Th22 cells detected following co-culture with PA infected DCs was similar in healthy controls and patients with CF (see Figure S2). However, although the observed IL-17A cytokine response was of a similar magnitude (Figure 4A), there was a significantly lower proportion of Th17 cells in CF patients compared with healthy controls (Figure 5D). This was particularly evident in response to the clinical PA strains, where median percentage of Th17 cells was 5.3% in healthy controls compared with 1.04% in CF patients (p = 0.003).

These cytokine profiles were in distinct contrast to those obtained from memory CD4^+^ T helper cells that were stimulated with either tetanus-toxoid treated DCs (Figure S3A, B) or heat-killed *Candida albicans* (Figure S3C, D). Tetanus-toxoid induced, as predicted, an essentially pure Th1 response. *Candida albicans*, as has been previously described [26], induced a robust Th17 response, although Th1 and Th22 cells were also evident.

### Tissue-homing characteristics of-specific memory Th22 and Th17 cells

**Figure 6:**
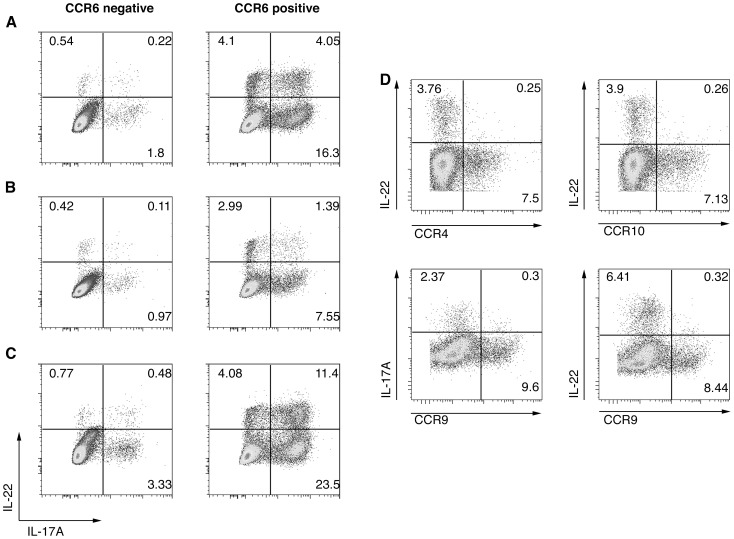
Tissue-homing characteristics of Pseudomonas aeruginosa-specific memory Th22 and Th17 cells. **A–C** Human memory CD4^+^ T cells were sorted into CCR6-depleted (CCR6 negative) and CCR6-enriched (CCR6 positive) populations before being exposed to dendritic cells (DCs) infected with Yorkhill 5 MOI30 (**A**), DCs infected with PA103 ΔUΔT MOI30 (**B**), or stimulated with anti-CD3 and anti-CD28 which acted as a positive control by polyclonal T cell stimulation (**C**). Plots show CD4^+^ IFN-γ negative T cells after 6-days of co-culture. **D** Memory CD4^+^ T cells were cultured with DCs infected with PA103 ΔpcrV MOI30. Pseudomonas-specific memory CD4^+^ T cells were analysed for expression of intracellular IL-22 or IL-17 as shown together with the skin-homing chemokine receptors CCR4, CCR10 and CCR9 as indicated. Numbers in each plot represent per cent cells in each quadrant. Results are representative of those seen in three independent individuals.

Human memory Th17 and Th22 cells characteristically express the mucosal chemokine receptor CCR6 [13], [16], [17]. To further verify the existence of memory Th17 and Th22 populations against PA, we sorted memory CD4^+^ T cells on the basis of their CCR6 expression prior to culture with PA-infected DCs. This confirmed that virtually all IFN-γ negative CD4^+^ T cell producing IL-17A and/or IL-22 (i.e. Th17 and Th22 cells) in response to PA infection were in the CCR6-enriched population (Figure 6A, B and C).

We next addressed whether the PA-specific memory Th17 and Th22 populations demonstrated features of homing to specific tissues. Th22 cells have previously been found to demonstrate a skin-homing phenotype [13], [16], [17] and have been implicated in the pathogenesis of inflammatory skin conditions [15]. We thus analysed the PA-specific memory IL-22 secreting cell population for expression of the skin-homing receptors CCR4 and CCR10. In contrast to the reported properties of peripheral blood derived Th22 cells without a characterised antigen specificity [16], [17], IL-22 production in response to PA infection was from memory CD4^+^ T cells that did not express the skin-homing receptors CCR4 or CCR10 (Figure 6D). Additionally, we found that PA-specific IL-17A and IL-22 expressing memory T helper cells did not display the gut-homing receptor CCR9 (Figure 6D).

## Discussion

The work described in this paper provides an important advance in our understanding of the human T helper response to *P. aeruginosa* and how this might influence the course of lung disease in individuals with CF who are chronically colonized with this microbe, as well as in other patients with PA infections. We found robust memory Th responses to *P. aeruginosa* in both normal individuals and in those with CF who have Pseudomonas colonization of the respiratory tract. These specific responses to *P. aeruginosa* contain Th1, Th17 and Th22 cells. This is the first time that an antigen-specific human Th22 response has been demonstrated. Moreover, these cells have a novel phenotype, lacking homing markers for skin and gut although retaining CCR6, which would allow them to respond to the ligands CCL20 and β-defensins produced from damaged epithelia. We show that although Th22 and Th1 responses are similar in controls and patients with CF, the proportion of Th17 responding cells is significantly diminished in peripheral blood of patients with CF. Taken together, these data show that chronic airway colonization with *P. aeruginosa* could activate both Th17 and Th22 memory responses that would migrate to inflamed lung, and potentially alter the balance between inflammation, host defence and tissue repair.

The most striking finding of this study was the identification of Pseudomonal-specific Th22 cells in both healthy individuals and in those with CF. We were surprised that normal healthy controls had robust Th22 (and indeed Th1 and Th17) responses to *P. aeruginosa*, as it is generally only pathogenic in immunocompromised states. The microbe is found within the bowel flora of a minority of normal individuals, 4% in one report [27], so it would not seem likely that this is the source of bacteria to provoke a memory Th response. Indeed, the Pseudomonal-specific Th22 cells we identified did not have gut or skin homing receptors (Fig 6). Although *P. aeruginosa* is a common environmental organism, it remains unclear at what site this microbe triggers a memory T helper response. Others have also demonstrated cross-reactivity of Th17 cells against different bacterial serotypes and fungi [28], [29]. We have not sought to define the antigenic component, of which there may be many, of *Pseudomonas aeruginosa* eliciting the observed Th17 and Th22 response, which might be shared with other infecting organisms and particularly with other Gram negative bacteria. The possibility of cross-reactivity of these Th cell responses remains, yet we have shown a lack of gut-homing and thus cross-reactivity with phylogenetically-related organisms in gut flora is unlikely.

In common with previous descriptions of Th22 cells in humans, the Pseudomonal-specific Th22 cells we identified were in the CCR6^+^ population (Figure 6) [15]–[17]. Previous studies identified these cells as bearing skin-homing markers and a pathogenic role for these cells in psoriasis and other inflammatory skin disorders has been proposed. We propose that the pseudomonal-specific Th22 cells we have identified in healthy humans and patients with CF can migrate to inflamed lung, which will release the chemokines CCL20 and β-defensins, both ligands for CCR6. In relation to pulmonary recruitment, we have previously sought evidence of Th17 and Th22 recruitment within bronchoalveolar lavage (BAL) from CF patients, an approach that has significant technical limitations. We have, however, identified low level IL-22 production (IL-22 detectable in 5 of 14 CF patients, with median IL-22 levels of 12.39pg/mL) within the CF BAL and thus further implicating a potential Th22 response within the disease pathogenesis. Given its potent action in promoting epithelial repair, regulating fibrosis and mediating host defense [14], [19], [30], we believe that IL-22 is important in attenuating the effects of chronic pulmonary infection with *P. aeruginosa* and enhancing tissue repair. The action of IL-22 may well be modified by IL-17, as demonstrated in lung fibrosis [30]. Testing of this hypothesis will best be performed in an animal model of chronic *P. aeruginosa* lung infection. We found in general that IL-22 production in the T cell memory population was predominantly from Th22 cells more than Th17 cells (Figures 2 and 6). As outlined above, these cells would be able to migrate to inflamed tissue producing the CCR6 ligands CCL20 and β-defensins, both of which would be found in infected lung. The swift expansion of a PA-specific Th22 memory cell population would thus ensure a rapid production of an IL-22 producing cell that could migrate to the site of infection to mediate host defence and/or contribute to tissue repair.

A very recent study from Jay Kolls' group identified Pseudomonal-specific Th17 cells in the draining lymph nodes of patients with CF [31]. Intriguingly, we found a reduction in Pseudomonal-specific Th17 cells in peripheral blood of CF patients. These data suggest that this Th17 population translocates from blood to pulmonary system during the chronic pulmonary colonization with *P. aeruginosa*. Indeed, IL-17^+^ T cells and IL-17 production has previously been observed within CF lung [10] and BAL [32], respectively. Alternatively, a recent study demonstrated that PA can induce myeloid-derived suppressor cells via flagellin stimulation [33]. These cells could act to down-regulate Th17 responses to PA, accounting for the observed reduction in PA-specific Th17 cells in CF patients.

Within the CF patient population exogenous and endogenous factors which may modulate immune function are common. For example, within our sampled CF population all patients were receiving long-term low-dose macrolide therapy and a quarter had CF-related diabetes mellitus. Nutritional status of CF patients will also be worse than age-matched controls. Controlling for such variables is problematic without a large sample size, which would be prohibitive for our described method of *ex vivo* T cell stimulation. We did however achieve adequate age matching of CF patients and controls, as well as ensuring that controls were healthy and free from conditions linked to pulmonary Th17 responses and pulmonary Pseudomonas infections, such as asthma and bronchiectasis. The data identifies and describes a previously uncharacterized Th cell response against *Pseudomonas aeruginosa* in both healthy controls and patients with CF, however generalizations to the whole heterogeneous CF population are preliminary at present.

Although this study has focussed on PA-specific Th cells in patients with cystic fibrosis, PA infections are significant in immunocompromised patients [34], in ventilator-associated pneumonia [35], following extensive burns and as a cause of infective exacerbations in patients with chronic obstructive airways disease [36]. Thus, the presence of Th22, Th17 and Th1 memory CD4^+^ cells specific to PA may play a role in these situations as well. The balance of effects on host defence, tissue repair and systemic inflammation produced by Il-22 will likely differ between these different conditions, necessitating further experimental investigation to define the role of IL-22 and Th22 cells in each of these conditions.

In conclusion, our work has demonstrated the presence in peripheral blood of memory CD4^+^ T cells of the Th22 lineage that are specific for *P. aeruginosa* antigens. They lack conventional skin or gut homing markers but are CCR6^+^. We propose that these cells will migrate to areas of inflamed lung under the influence of CCL20 or β-defensins. Local IL-22 within the lung could play a key role in host defence, epithelial repair and regeneration, and may be amenable to therapeutic intervention to promote healthy lung repair after damaging infection.

## Supporting Information

Figure S1 **Cytokine production by human memory CD4^+^ T cells to different** ***Pseudomonas aeruginosa*** **strains.** Human memory CD4^+^ T cells from healthy volunteers (**A**, **B** and **C**) and patients with cystic fibrosis (CF) (**D**, **E** and **F**) were co-cultured with dendritic cells infected with different strains of (PA). Laboratory PA strains PA103 ΔpcrV and PA103 ΔUΔT, clinical non-mucoid strains Yorkhill 1 and 2, and the clinical mucoid strain Yorkhill 5 were used; clinical strains were derived from cystic fibrosis patients. Levels of cytokines were measured in the supernatant after 6-days of culture. Each point represents the result from one individual; an individual may be represented more than once by separate experiments. The line indicates the median value. Differences between strains were evaluated by a Kruskal-Wallis (KW) test with pairwise assessments of differences between groups made using Dunn's multiple comparison test. *, significance difference <0.05. Only minor differences were seen in the cytokine response to different strains of PA in both healthy controls and patients with CF.
(TIF)

Figure S2 **Memory CD4^+^ T cells subset response to different Pseudomonas strains in CF and controls.** The proportion of PA specific CD4^+^ T helper cell responses to PA103 (PA103 ΔpcrV and PA103 ΔUΔT) and Yorkhill strains (Yorkhill 1, 2 and 5) that are Th1 (**A**) or Th22 (**B**) was determined as outlined in Figure 5 in the main text. Each point represents the result from one individual; some individuals were tested against different bacterial strains as indicated by the symbols used in Figure 4. The line indicates the median value. Differences between controls and patients with CF were evaluated by a Mann-Whitney test with the p value as shown.
(TIF)

Figure S3 **Cytokine production by human memory CD4^+^ T cells to tetanus toxoid and heat-killed candida albicans.** Human memory CD4^+^ T cells from healthy were co-cultured with dendritic cells infected with tetanus toxoid or heat-killed candida albicans. (**A**) Patterns of cytokine expression by non-proliferating CD4^+^ T cells following 6-days of culture with DCs infected with tetanus toxoid. (**B**) Patterns of cytokine expression by CD4^+^ T cells proliferating in response to culture with DCs infected with tetanus toxoid. (**C**) Patterns of cytokine expression by non-proliferating CD4^+^ T cells following 6-days of culture with DCs infected with heat-killed candida albicans (HKCA). (**D**) Patterns of cytokine expression by CD4^+^ T cells proliferating in response to culture with DCs infected with HKCA. Numbers in plot represent percent cells in each quadrant.
(TIF)

## References

[1] Rowe SM, Miller S, Sorscher EJ (2005) Cystic fibrosis. N Engl J Med 352: 1992–2001. 15888700 10.1056/NEJMra043184

[2] Ratjen F, McColley SA (2012) Update in cystic fibrosis 2011. Am J Respir Crit Care Med 185: 933–936. 22550209 10.1164/rccm.201202-0306UP

[3] Sadikot RT, Blackwell TS, Christman JW, Prince AS (2005) Pathogen-host interactions in Pseudomonas aeruginosa pneumonia. Am J Respir Crit Care Med 171: 1209–1223. 15695491 10.1164/rccm.200408-1044SO PMC2718459

[4] Dubin PJ, Kolls JK (2007) Pseudomonas aeruginosa and the host pulmonary immune response. Expert Rev Respir Med 1: 121–137. 20477272 10.1586/17476348.1.1.121

[5] Downey DG, Bell SC, Elborn JS (2009) Neutrophils in cystic fibrosis. Thorax 64: 81–88. 19103874 10.1136/thx.2007.082388

[6] Doring G (1989) Host response to Pseudomonas aeruginosa. Acta Paediatr Scand Suppl 363: 37–39; discussion 40. 10.1111/apa.1989.78.s363.37 2518396

[7] Markham RB, Powderly WG (1988) Exposure of mice to live Pseudomonas aeruginosa generates protective cell-mediated immunity in the absence of an antibody response. J Immunol 140: 2039–2045. 3126239

[8] Stockinger B, Veldhoen M, Martin B (2007) Th17 T cells: linking innate and adaptive immunity. Semin Immunol 19: 353–361. 18023589 10.1016/j.smim.2007.10.008

[9] Priebe GP, Walsh RL, Cederroth TA, Kamei A, Coutinho-Sledge YS, et al (2008) IL-17 is a critical component of vaccine-induced protection against lung infection by lipopolysaccharide-heterologous strains of Pseudomonas aeruginosa. J Immunol 181: 4965–4975. 18802100 10.4049/jimmunol.181.7.4965 PMC2597098

[10] Tan HL, Regamey N, Brown S, Bush A, Lloyd CM, et al (2011) The Th17 pathway in cystic fibrosis lung disease. Am J Respir Crit Care Med 184: 252–258. 21474644 10.1164/rccm.201102-0236OC PMC3381840

[11] Brodlie M, Corris PA, Lordan J, Ward C (2012) Interleukin-17 and cystic fibrosis lung disease. Am J Respir Crit Care Med 185: : 108–109; author reply 109–110. 10.1164/ajrccm.185.1.108a 22210793

[12] Zielinski CE, Mele F, Aschenbrenner D, Jarrossay D, Ronchi F, et al (2012) Pathogen-induced human TH17 cells produce IFN-gamma or IL-10 and are regulated by IL-1beta. Nature 484: 514–518. 22466287 10.1038/nature10957

[13] Acosta-Rodriguez EV, Rivino L, Geginat J, Jarrossay D, Gattorno M, et al (2007) Surface phenotype and antigenic specificity of human interleukin 17-producing T helper memory cells. Nat Immunol 8: 639–646. 17486092 10.1038/ni1467

[14] Wolk K, Witte E, Witte K, Warszawska K, Sabat R (2010) Biology of interleukin-22. Semin Immunopathol 32: 17–31. 20127093 10.1007/s00281-009-0188-x

[15] Eyerich S, Eyerich K, Pennino D, Carbone T, Nasorri F, et al (2009) Th22 cells represent a distinct human T cell subset involved in epidermal immunity and remodeling. J Clin Invest 119: 3573–3585. 19920355 10.1172/JCI40202 PMC2786807

[16] Duhen T, Geiger R, Jarrossay D, Lanzavecchia A, Sallusto F (2009) Production of interleukin 22 but not interleukin 17 by a subset of human skin-homing memory T cells. Nat Immunol 10: 857–863. 19578369 10.1038/ni.1767

[17] Trifari S, Kaplan CD, Tran EH, Crellin NK, Spits H (2009) Identification of a human helper T cell population that has abundant production of interleukin 22 and is distinct from T(H)-17, T(H)1 and T(H)2 cells. Nat Immunol 10: 864–871. 19578368 10.1038/ni.1770

[18] Basu R, O'Quinn DB, Silberger DJ, Schoeb TR, Fouser L, et al (2012) Th22 cells are an important source of IL-22 for host protection against enteropathogenic bacteria. Immunity 37: 1061–1075. 23200827 10.1016/j.immuni.2012.08.024 PMC3678257

[19] Aujla SJ, Chan YR, Zheng M, Fei M, Askew DJ, et al (2008) IL-22 mediates mucosal host defense against Gram-negative bacterial pneumonia. Nat Med 14: 275–281. 18264110 10.1038/nm1710 PMC2901867

[20] Ota N, Wong K, Valdez PA, Zheng Y, Crellin NK, et al (2011) IL-22 bridges the lymphotoxin pathway with the maintenance of colonic lymphoid structures during infection with Citrobacter rodentium. Nat Immunol 12: 941–948. 21874025 10.1038/ni.2089

[21] Zheng Y, Valdez PA, Danilenko DM, Hu Y, Sa SM, et al (2008) Interleukin-22 mediates early host defense against attaching and effacing bacterial pathogens. Nat Med 14: 282–289. 18264109 10.1038/nm1720

[22] Sonnenberg GF, Monticelli LA, Alenghat T, Fung TC, Hutnick NA, et al (2012) Innate lymphoid cells promote anatomical containment of lymphoid-resident commensal bacteria. Science 336: 1321–1325. 22674331 10.1126/science.1222551 PMC3659421

[23] Vallis AJ, Finck-Barbancon V, Yahr TL, Frank DW (1999) Biological effects of Pseudomonas aeruginosa type III-secreted proteins on CHO cells. Infect Immun 67: 2040–2044. 10085057 10.1128/iai.67.4.2040-2044.1999 PMC96567

[24] Sawa T, Yahr TL, Ohara M, Kurahashi K, Gropper MA, et al (1999) Active and passive immunization with the Pseudomonas V antigen protects against type III intoxication and lung injury. Nat Med 5: 392–398. 10202927 10.1038/7391

[25] Gurney AL (2004) IL-22, a Th1 cytokine that targets the pancreas and select other peripheral tissues. Int Immunopharmacol 4: 669–677. 15120651 10.1016/j.intimp.2004.01.016

[26] Gaffen SL, Hernandez-Santos N, Peterson AC (2011) IL-17 signaling in host defense against Candida albicans. Immunol Res 50: 181–187. 21717069 10.1007/s12026-011-8226-x PMC3257840

[27] Stoodley BJ, Thom BT (1970) Observations on the intestinal carriage of Pseudomonas aeruginosa. J Med Microbiol 3: 367–375. 4990646 10.1099/00222615-3-3-367

[28] Wuthrich M, Hung CY, Gern BH, Pick-Jacobs JC, Galles KJ, et al (2011) A TCR transgenic mouse reactive with multiple systemic dimorphic fungi. J Immunol 187: 1421–1431. 21705621 10.4049/jimmunol.1100921 PMC3140549

[29] Chen K, McAleer JP, Lin Y, Paterson DL, Zheng M, et al (2011) Th17 cells mediate clade-specific, serotype-independent mucosal immunity. Immunity 35: 997–1009. 22195749 10.1016/j.immuni.2011.10.018 PMC3406408

[30] Sonnenberg GF, Nair MG, Kirn TJ, Zaph C, Fouser LA, et al (2010) Pathological versus protective functions of IL-22 in airway inflammation are regulated by IL-17A. J Exp Med 207: 1293–1305. 20498020 10.1084/jem.20092054 PMC2882840

[31] Chan YR, Chen K, Duncan SR, Lathrop KL, Latoche JD, et al.. (2012) Patients with cystic fibrosis have inducible IL-17(+)IL-22(+) memory cells in lung draining lymph nodes. J Allergy Clin Immunol. 10.1016/j.jaci.2012.05.036 PMC3488163 22795370

[32] Brodlie M, McKean MC, Johnson GE, Anderson AE, Hilkens CM, et al (2011) Raised interleukin-17 is immunolocalised to neutrophils in cystic fibrosis lung disease. Eur Respir J 37: 1378–1385. 21109552 10.1183/09031936.00067110

[33] Rieber N, Brand A, Hector A, Graepler-Mainka U, Ost M, et al (2013) Flagellin induces myeloid-derived suppressor cells: implications for Pseudomonas aeruginosa infection in cystic fibrosis lung disease. J Immunol 190: 1276–1284. 23277486 10.4049/jimmunol.1202144

[34] Pier GB, Ramphal R (2005) Pseudomonas aeruginosa. In: Mandell GL, Bennett JE, Dolin R, editors. Principles and Practice of Infectious Diseases. 6th ed. Philadelphia: Elsevier Churchill Livingstone. pp. 2587–2615.

[35] Wunderink RG (2005) Nosocomial pneumonia, including ventilator-associated pneumonia. Proc Am Thorac Soc 2: 440–444. 16322597 10.1513/pats.2005080-83JS

[36] Martinez-Solano L, Macia MD, Fajardo A, Oliver A, Martinez JL (2008) Chronic Pseudomonas aeruginosa infection in chronic obstructive pulmonary disease. Clin Infect Dis 47: 1526–1533. 18990062 10.1086/593186

