# Supplementary figures and images for: T Helper Cell Subsets Specific for Pseudomonas aeruginosa in Healthy Individuals and Patients with Cystic Fibrosis
Source: PLoS One. 2014 Feb 27;9(2):e90263. doi: 10.1371/journal.pone.0090263 (PMC3937364; doi:10.1371/journal.pone.0090263)

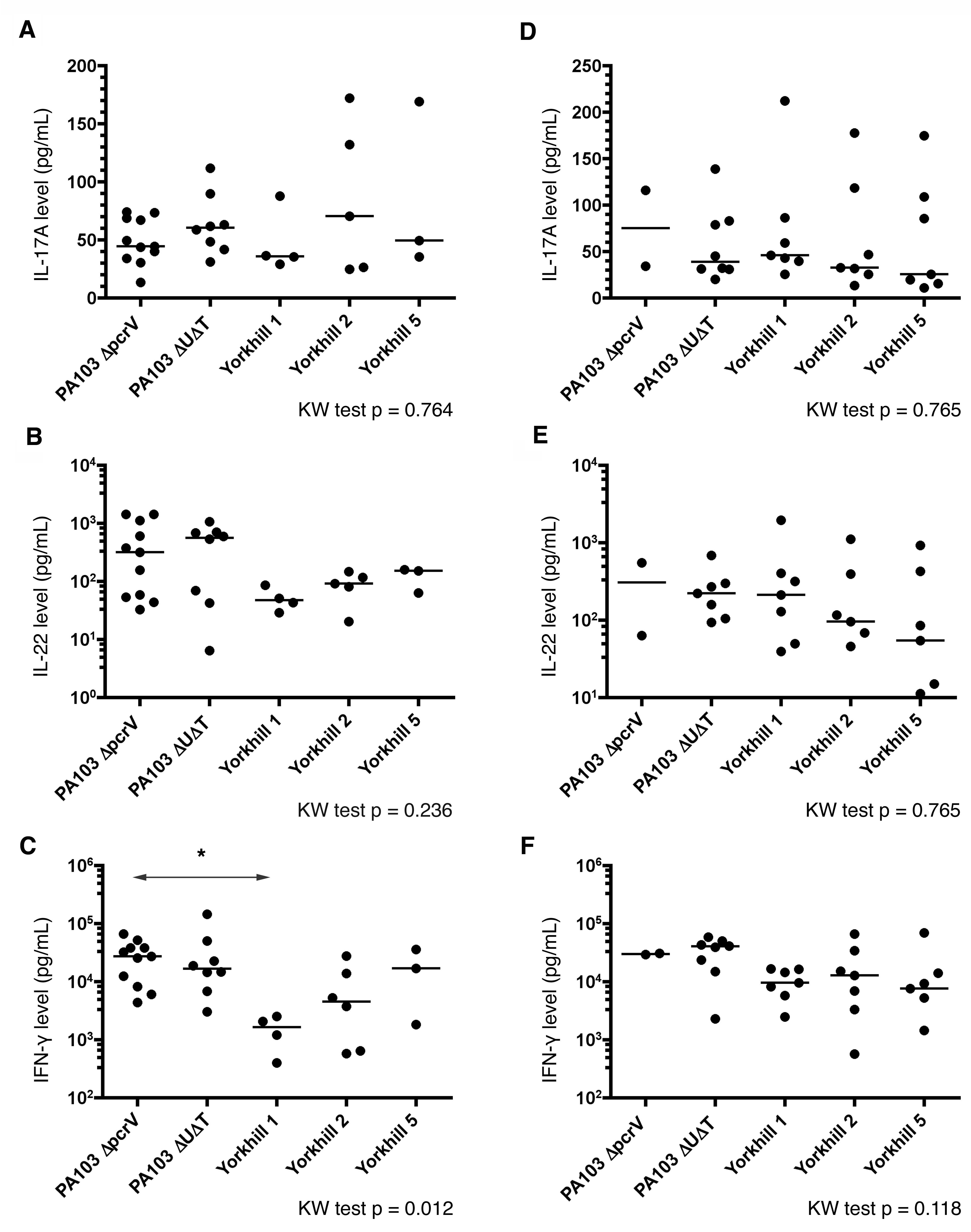

Supplement: Figure S1 — Cytokine production by human memory CD4+ T cells to different Pseudomonas aeruginosa strains. Human memory CD4+ T cells from healthy volunteers (A, B and C) and patients with cystic fibrosis (CF) (D, E and F) were co-cultured with dendritic cells infected with different strains of (PA). Laboratory PA strains PA103 ΔpcrV and PA103 ΔUΔT, clinical non-mucoid strains Yorkhill 1 and 2, and the clinical mucoid strain Yorkhill 5 were used; clinical strains were derived from cystic fibrosis patients. Levels of cytokines were measured in the supernatant after 6-days of culture. Each point represents the result from one individual; an individual may be represented more than once by separate experiments. The line indicates the median value. Differences between strains were evaluated by a Kruskal-Wallis (KW) test with pairwise assessments of differences between groups made using Dunn's multiple comparison test. *, significance difference <0.05. Only minor differences were seen in the cytokine response to different strains of PA in both healthy controls and patients with CF. (TIF) [file pone.0090263.s001.tif]

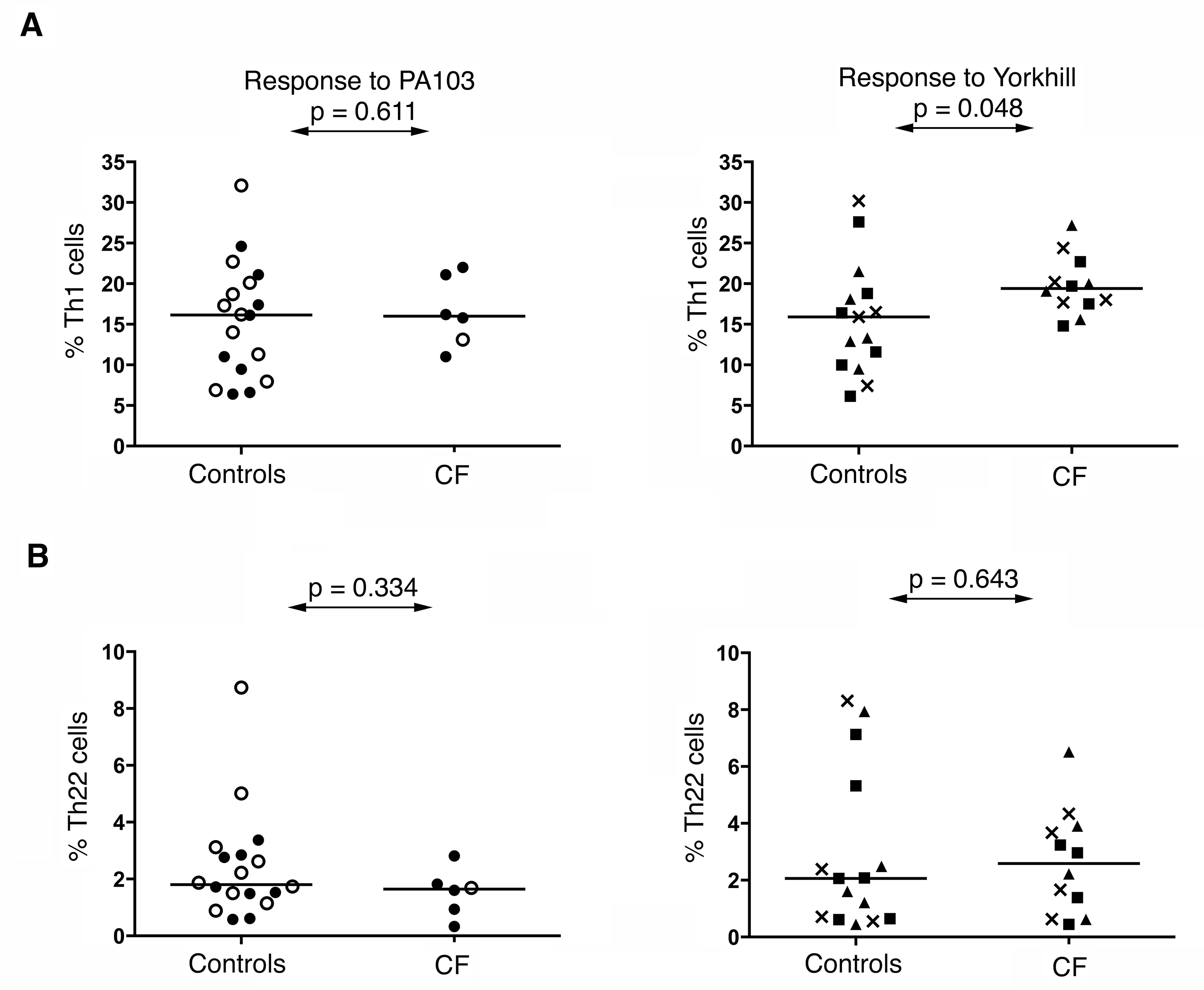

Supplement: Figure S2 — Memory CD4+ T cells subset response to different Pseudomonas strains in CF and controls. The proportion of PA specific CD4+ T helper cell responses to PA103 (PA103 ΔpcrV and PA103 ΔUΔT) and Yorkhill strains (Yorkhill 1, 2 and 5) that are Th1 (A) or Th22 (B) was determined as outlined in Figure 5 in the main text. Each point represents the result from one individual; some individuals were tested against different bacterial strains as indicated by the symbols used in Figure 4. The line indicates the median value. Differences between controls and patients with CF were evaluated by a Mann-Whitney test with the p value as shown. (TIF) [file pone.0090263.s002.tif]

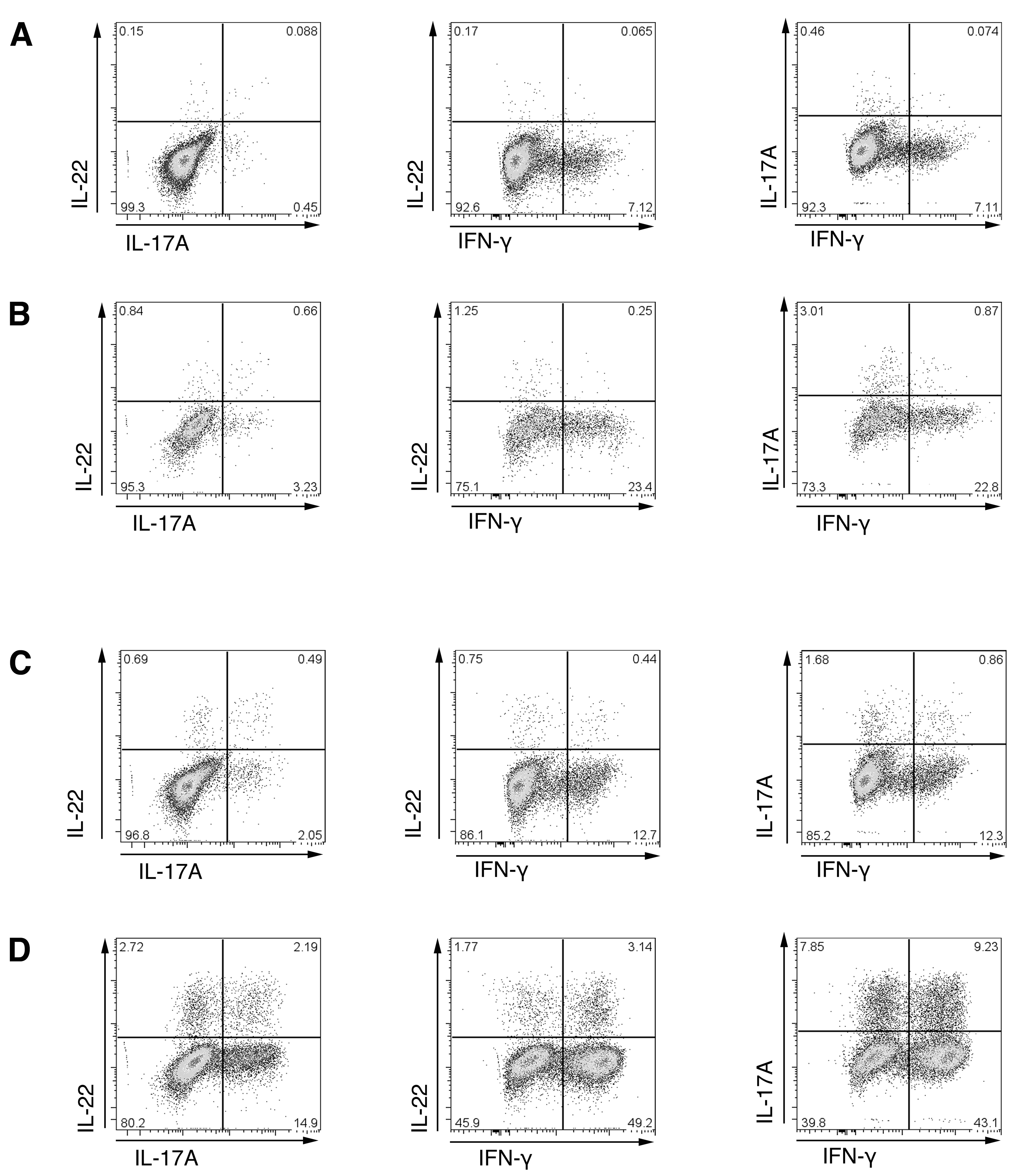

Supplement: Figure S3 — Cytokine production by human memory CD4+ T cells to tetanus toxoid and heat-killed candida albicans. Human memory CD4+ T cells from healthy were co-cultured with dendritic cells infected with tetanus toxoid or heat-killed candida albicans. (A) Patterns of cytokine expression by non-proliferating CD4+ T cells following 6-days of culture with DCs infected with tetanus toxoid. (B) Patterns of cytokine expression by CD4+ T cells proliferating in response to culture with DCs infected with tetanus toxoid. (C) Patterns of cytokine expression by non-proliferating CD4+ T cells following 6-days of culture with DCs infected with heat-killed candida albicans (HKCA). (D) Patterns of cytokine expression by CD4+ T cells proliferating in response to culture with DCs infected with HKCA. Numbers in plot represent percent cells in each quadrant. (TIF) [file pone.0090263.s003.tif]
